# Poly(vinylbenzylchloride) Based Anion-Exchange Blend Membranes (AEBMs): Influence of PEG Additive on Conductivity and Stability

**DOI:** 10.3390/membranes7020032

**Published:** 2017-06-16

**Authors:** Jochen A. Kerres, Henning M. Krieg

**Affiliations:** 1 Institute of Chemical Process Engineering, University of Stuttgart, 70199 Stuttgart, Germany; 2 Faculty of Natural Science, North-West University, Focus Area: Chemical Resource Beneficiation, Potchefstroom 2520, South Africa; henning.krieg@nwu.ac.za

**Keywords:** anion-exchange blend membrane, poly(vinylbenzylchloride), polybenzimidazole, ionic cross-link, Cl^−^ conductivity, impedance spectroscopy, TGA-FTIR coupling

## Abstract

In view of the many possible applications such as fuel cells and electrolysers, recent interest in novel anion exchange membranes (AEMs) has increased significantly. However, their low conductivity and chemical stability limits their current suitability. In this study, the synthesis and characterization of several three- and four-component anion exchange blend membranes (AEBMs) is described, where the compositions have been systematically varied to study the influence of the AEBM’s composition on the anion conductivities as well as chemical and thermal stabilities under strongly alkaline conditions. It was shown that the epoxide-functionalized poly(ethylene glycol)s that were introduced into the four-component AEBMs resulted in increased conductivity as well as a marked improvement in the stability of the AEBMs in an alkaline environment. In addition, the thermal stability of the novel AEBMs was excellent showing the suitability of these membranes for several electrochemical applications.

## 1. Introduction

Over the past decades, the research interest in anion exchange membranes (AEMs) for electrochemical conversion processes has increased significantly with possible applications of AEMs in alkaline polymer electrolyte fuel cells (APEFCs) [1], alkaline polymer electrolyte electrolysis (APEE) [2], redox flow batteries (RFBS) [3], reverse electrodialysis (RED) [4] and bio-electrochemical systems, including microbial fuel cells (MFC) [5] and enzymatic fuel cells [6]. In addition, AEMs are used in electrodialysis (ED) [7] and Donnan Dialysis [8]—or diffusion dialysis (DD) [9]. A significant advantage of using AEMs in electrochemical conversion processes such as fuel cells or electrolysis is that no noble metal catalysts (consisting of platinum group metals (PGM)) are required for the electrocatalytic reactions at the electrodes, as was shown by Piana et al. [1], which implies that AEM containing membrane electrode assemblies (MEAs) are potentially much cheaper than cation exchange membranes (CEM) containing MEAs. However, AEMs have, compared to CEM, the following disadvantages:

- The ionic conductivity of AEMs is significantly lower than that of CEMs despite having comparable ion exchange capacity (IEC), which is partly due to the fact that most AEM types have a hydrocarbon backbone, which is significantly less hydrophobic than, for example, the perfluorinated polymer main chain of the perfluorinated membranes of the Nafion^®^ (Fayetteville, NC, USA) type, leading to a smaller separation between ionic groups and the polymer backbone and, therefore, to lower ionic conductivities due to the lower local density of the anion-exchange groups, particularly since in most AEM types the cationic head groups are connected to the polymer backbone only via a CH_2_ (benzylic) bridge [10] which hinders clustering of the anion-exchange groups.
- If the AEMs are exchanged specifically with OH^−^ counterions, their chemical stability is limited when used in APEFCs or APEEs, since the OH^−^ counterion can degrade the cationic head group [11] or even the polymer backbone [12].

Most current research aims at minimizing these disadvantages of AEMs thereby improving their properties. The starting polymers for commonly used AEM polymers are aromatic group-containing polymers, such as polystyrene, polyphenylene ether, or other aromatic polyethers such as polyethersulfones or polyether ketones which are substituted with methyl groups. The first step for the preparation of an AEM is the introduction of halomethyl groups into the polymer. This halomethylation is achieved by either: (1) bromomethylation with hydrogen halide, formaldehyde and a Lewis acid such as ZnCl_2_ or AlCl_3_ (Blanc reaction [13,14]); or (2) bromination of the –CH_3_ side groups of aromatic polymers using *N*-bromo-succinimide (NBS) and a radical starter as the bromination agent (Wohl–Ziegler reaction [15]). Since the Blanc reaction includes the strongly carcinogenic intermediate *bis*(chloromethyl) ether, the Wohl–Ziegler reaction is currently preferably used for the synthesis of halomethylated aromatic polymers. Literature examples of the preparation of bromomethylated aromatic polymers by the Wohl–Ziegler reaction are the bromomethylation of poly(phenylene oxide) [16], or methylated poly(ether sulfone) [17]. The conversion of the –CH_2_Hal group (Hal = Cl, Br) to an anion exchange group can be attained by the reaction with a tertiary amine such as trimethylamine [14], pyridine [18], pentamethylguanidine [19] or an *N*-alkylated (*benz*)imidazole [20]. The conductivity of AEMs can be increased by increasing the distance between the polymer backbone and the cationic head groups thereby obtaining a greater local concentration of ion-conducting groups. Phase-segregated AEMs with improved ionic conductivity can be achieved by preparing linear (multi) block copolymers composed of hydrophobic and ion-containing blocks [21] or graft copolymers with an anion-exchange graft side chain [22] (for example, by the grafting of vinyl benzyl side chains onto an e^−^-irradiated ETFE (ethylene-tetrafluoroethylene copolymer) film and by quaternizing the chloromethylated side chains with trimethylamine [23]). Another promising approach to improve the distance between the backbone and cationic group, to facilitate the clustering of anion-exchange groups to increase the anion conductivity, is to place the anion-exchange groups either within or at the end of an aliphatic side chain or both. For example, Dang and Jannasch have recently prepared novel PPO AEMs by a four-step procedure [24]: First, PPO was lithiated at the methyl groups, followed by quenching with an excess of a dihalogenoalkane, e.g., dibromohexane. The formed PPO-AEMs with the halomethyl group at the end of the aliphatic side chain were then reacted with an excess of a hexanediamine, e.g. N,N,N’,N’-tetramethyldiaminohexane, affording AEMs with the cationic group between the first and the second aliphatic group. The tertiary amino group remaining at the end of the side chain was then quaternized with CH_3_I, ending up with AEMs containing two ammonium groups in the side chain. These AEMs showed the expected high anion conductivity and, surprisingly, excellent chemical stabilities in an alkaline environment which was ascribed to the absence of chemically labile benzylic links in these AEMs. Hickner et al. used a similar multication approach for the synthesis of side-chain AEMs based on PPO. Similar to those of the Jannasch group, the membranes were highly conductive (up to 80 mS·cm^−1^) and remarkably stable (15%–50% conductivity loss after 20 days in 1 M NaOH at 80 °C) [25]. In order to achieve an improvement of the chemical stability of AEMs, the combination of the anion exchange groups and the polymer main chain must be studied as the stability of the anion exchange always also depends on the polymeric main chain. For example, it was shown that a benzyltrimethylammonium (BTMA) head group bound to a poly(styrene) backbone shows slightly better chemical stability in an alkaline medium (0.6 M KOH, 80 °C) than BTMA bound to PPO and a much higher chemical stability than BTMA bound to a poly(phenylene ether sulfone (PES)) backbone [12]. It is obviously not easy to predict which polymer backbone is stable, which can be seen from the above example, since all three polymers (PSt, PPO and PES) contain aromatic groups of comparable electron density. However, it has been found that the alkali stability of the cationic head groups of AEMs can be significantly improved by sterically shielding the anion-exchange groups from nucleophilic OH^−^ counterion attack. In a study by Thomas et al., two different polybenzimidazolium- (PBIm^+^) based AEMs were studied in terms of their stability in an alkaline environment [26]. One of the PBIm^+^-AEMs contained methyl groups adjacent to the dimethylbenzimidazolium cation, the other not. While the sterically hindered PBIm^+^-AEM showed high stability in 2 M KOH, the unhindered one was rapidly degraded. The high stability of the hindered PBIm^+^-AEM was explained as follows: at the sterically hindered PBIm^+^-AEM, the OH^−^ group cannot attack the imidazolium ring. At the unhindered PBIm^+^-AEM, the OH^−^ group can attack the imidazolium ring leading to ring opening [27]. From these results, it is clear that shielding the anion-exchange groups from a possible OH^−^ attack by introducing bulky groups in the vicinity of the anion-exchange group is a promising concept. Other groups have also synthesized AEMs where the cationic group is surrounded by bulky functional groups. For example, Liu et al. synthesized sterically highly hindered PPO AEMs containing 1,4,5-trimethyl-2-(2,4,6-trimethoxyphenyl) imidazolium head groups, which possessed excellent alkaline stabilities (no decrease of ion exchange capacity (IEC) after 25 h storage in 1 M KOH at 80 °C [28]). Contrary, dimethylimidazolium group-modified PPO showed a strong IEC decrease (approximately 50% in 2 M KOH at 60 °C) after nine days [29]. Other strategies to reduce the chemical degradation of AEMs include:

- search for alternative fixed cations;
- chemical and/or physical cross-linking; and
- embedding of the anion-exchange polymers in an inert polymer matrix.

As an alternative to the most commonly used trialkylammonium cationic groups, the already mentioned pentamethylguanidinium group (PMG) has been introduced. However, it was found that the PMG cations are only chemically stable when they are resonance-stabilized, i.e., the positive charge of the PMG-cation is delocalized, which is the case when the PMG group is attached to an aromatic group which should preferably be electron-deficient, as shown by Kim et al. [30,31]. Another example of a cationic functional group chemically stabilized by steric hindrance is the tris (2,4,6-trimethoxyphenyl) phosphonium cation, which was bound to polyvinylbenzyl-graft chains [32], exhibiting no degradation after 75 h of storage in 1 N NaOH at 60 °C. In a study by Zha et al., a positively charged *bis*(terpyridine) ruthenium (II) complex was attached to a norbornene polymer [33]. This AEM exhibited excellent stability in alkaline environment as immersion of the polymer in 1 N NaOH at room temperature showed no degradation even after half a year. Another option of stabilizing AEMs is crosslinking. He et al. reported the synthesis of mechanically robust PPO-based AEMs which have been covalently cross-linked (via quaternization) in a multistage process with tertiary diamines and vinyl benzyl chloride [34]. In a study by Cheng et al. chloromethylated PSU was cross-linked (via quaternization) with the new N-basic difunctional reagent guanidimidazol. These novel crosslinked polymers had better stability to alkali than similar AEMs, which have been quaternized with 1-methylimidazole without cross-linking [35].

In our group, bromomethylated PPO was quaternized with the diamine DABCO and 1.4-diiodobutane and embedded in the matrix polymer PVDF. Mechanically and chemically stable covalently crosslinked AEMs were obtained, which exhibited no degradation (IEC and conductivity) even after 10 days of storage in 1 N KOH at 90 °C, while yielding a high performance in direct methanol fuel cells (DMFC) (4 M MeOH and 5 M KOH) [36]. In another study methylated PBIOO obtained from Fuma-Tech (Bietigheim-Bissingen, Germany), which had been prepared using a novel non-carcinogenic reagent, was blended with sulfonated PSU and covalently crosslinked (quaternized) using DABCO and 1.4-diiodobutane [37]. In a DMFC using non-platinum catalysts (anode: 6% Pd/CeO_2_/C, cathode: 4% FeCo/C) these AEMs at 80 °C (anode Feed 4 M MeOH + 5 M KOH) performed comparable to that of a commercial Tokuyama-AEM (maximum power density 120 mW/cm^2^). In another study from our group, ionic and covalently crosslinked AEM blends were synthesized with bromomethylated PPO or a bromomethylated and partially fluorinated arylene main-chain polymer and a partially fluorinated PBI (F_6_PBI) as the mechanically and chemically stable matrix and sulfonated polyethersulfone sPPSU as the ionic macromolecular cross-linker. The halomethylated blend component was quaternized with *N*-methylmorpholine (NMM) [38]. Due to the interaction of the sulfonate groups of the sulfonated polymer and the basic *N*-methylmorpholinium cations, ionic crosslinking sites were formed, leading to an improved mechanical and chemical stability of the AEM blends. Covalent cross-links in the membrane matrix were formed during evaporation of the solvent by the reaction of a minor portion of the –CH_2_Cl groups of the PVBCl with some of the N–H-functional groups of the PBI blend components [39]. The alkaline stability of the membranes was investigated in 1 M KOH at 90 °C over a period of 10 days and compared to a commercial AEM of Tokuyama (A201). The most stable of the manufactured AEM blends lost about 40% of their initial Cl^−^ conductivity, while the commercial A201 possessed only 21% of the initial conductivity after this time period. Similar AEM blends were synthesized in a further study where NBS-brominated PPO was blended with PBIOO or F_6_PBI as the matrix polymer, while sPPSU was added as ionic cross-linker to the blend of brominated PPO and F_6_PBI. The quaternization of bromomethylated PPO leading to the anion-exchange groups was attained with 1-methylimidazole or 1-ethyl-3-methylimidazole [40]. After the stability tests (1 M KOH, 90 °C, 10 days), the blend membrane of 1-methylimidazole-quaternized PPO, F_6_PBI and sPPSU showed a conductivity of 69% of the original conductivity, while blends from PBIOO and PPO quaternized with the two imidazoles mentioned above exhibited a residual ion conductivity which was 31–43% of the original value.

A further interesting new approach was suggested by Hickner et al. where they synthesized mechanically strong and chemically stable AEMs from rigid-flexible semi-interpenetrating networks with trimethylammonium PPO as the rigid compound and a flexible network built up from poly(ethylene glycol)s (PEGs). These membranes possessed, probably due to the high hydrophilicity of PEG, high OH^−^ conductivities (up to 75 mS·cm^−1^), and good alkaline stabilities (conductivity decreased 25%–61% after 30 days at 80 °C in 1 M NaOH) [41].

**Figure 1:**
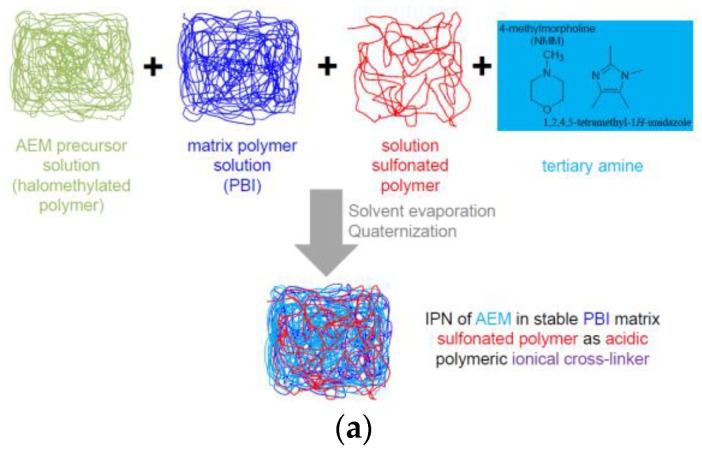

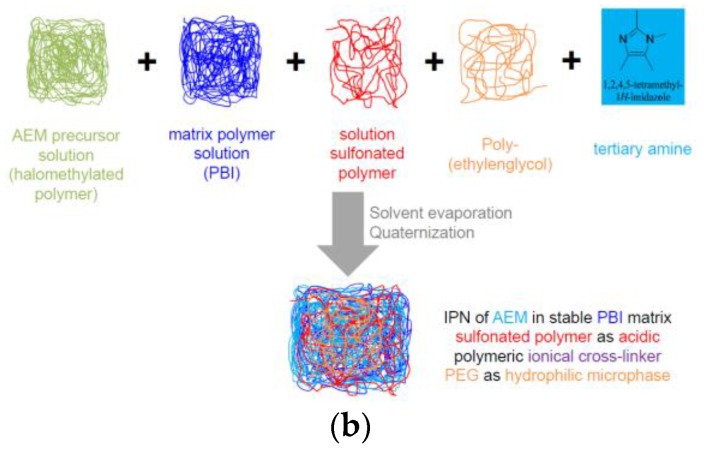
Preparation schemes for the: (**a**) three-component; and (**b**) four-component AEBMs.

**Figure 2:**
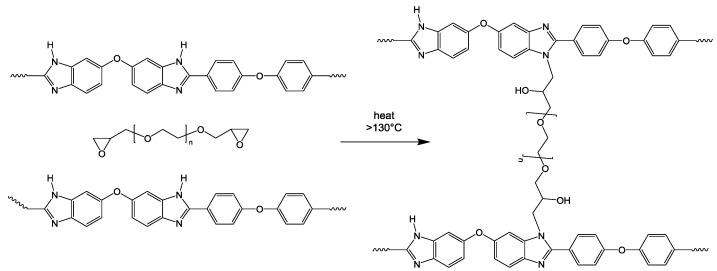
Reaction of epoxide end groups of PEGs with the imidazole groups of PBI.

In this study, several of the approaches described above were combined resulting in two types of novel anion-exchange blend membranes (AEBMs). Poly(vinylbenzylchloride) (PVBCl), was used as the AEM precursor, as this polymer is known to form stable AEMs [12], while different sterically hindered tertiary N-basic compounds were used for the quaternization of the PVBCl. The two novel AEBMs consisted of:

(a) Three-component blends (after quaternization of the chlormethyl groups with the tertiary N-basic compounds) consisting of PVBCl, a sterically hindered tertiary N base, the polybenzimidazoles PBIOO or F_6_PBI, and a nonfluorinated and a partially fluorinated aromatic sulfonated polyether as ionic cross-linker (Figure 1a);
(b) Four-component blends (after quaternization of the chlormethyl groups with the tertiary N-basic compounds) consisting of PVBCl, a sterically hindered tertiary N base, PBIOO, a nonfluorinated aromatic sulfonated polyethersulfone polymer as ionic cross-linker, and poly(ethylenglycol)s (PEGs) with different chain lengths and epoxide groups at the end of the PEG chain for anchoring in the PBI matrix by reacting the epoxy groups with the N–H groups of the imidazole moiety (Figure 1b). The reaction of the epoxy end groups of the PEG with the N-H groups of the PBI blend component is illustrated in Figure 2.

**Figure 3:**
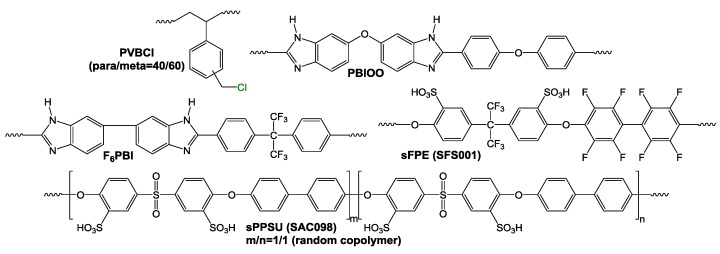
Polymers used for the AEBMs.

Figure 3 shows the polymers used for the preparation of the AEBMs.

In this study, the synthesis and characterization of several three- and four-component AEBMs is described, where the compositions were systematically varied to study the influence of the AEMBs composition on the resulting properties. AEBMs prepared from nonfluorinated blend components (PBIOO and sPPSU) were compared to those containing partially fluorinated polymeric compounds (F_6_PBI and sFPE (FPE = fluorinated polyether)). In the framework of the investigations, a particular focus was to study the influence of the AEBM composition on the anion conductivities and chemical and thermal stabilities under strongly alkaline conditions.

## 2. Results and Discussion

### 2.1. Membrane Properties

#### 2.1.1. Brief Discussion of the Physico-Chemical Properties of the AEBMs

In this study, three- and four-component AEBMs using poly(vinylbenzylchloride) as the anion-exchange precursor polymer were prepared, where the composition was systematically varied in terms of the blend component type and mass ratio. Most membranes were prepared using TMIm as the tertiary N compound as this sterically hindered methylated imidazole was found to be more stable in alkaline environments than other imidazoles that were used in an earlier study [40]. For comparative purposes, two AEBMs quaternized with PMP (2256) and NMM (2257), respectively, were also added to this study.

**Figure 4:**
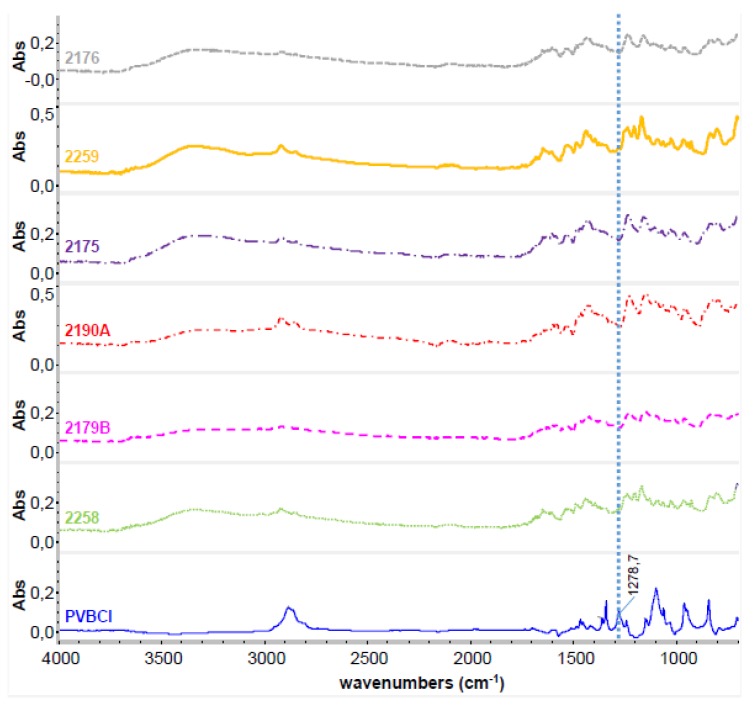
FTIR spectra of PVBCl and several of the prepared AEBMs.

Before varying the composition of the blend membranes, the extent of quaternization of the CH_2_Cl groups of poly(vinylbenzylchloride) (PVBCl) with TMIm was investigated by Attenuated Total Reflection Fourier-Transform Infrared Spectroscopy (ATR-FTIR). According to the literature, the stretching vibrations of the CH_2_Cl group of PVBCl are found in the vicinity of 675, 709 and 1267 cm^−1^ [42]. Since the band around 1267 cm^−1^ is very strong, this band was selected as indication of unreacted CH_2_Cl groups in the AEBMs. In Figure 4, the ATR-FTIR spectra of several of the AEBMs are compared to that of PVBCl (in this study, the CH_2_Cl band was found at 1279 cm^−1^), clearly indicating that no unreacted CH_2_Cl groups were present in the AEBMs.

The membrane compositions were varied in terms of: (a) the nonfluorinated/partially fluorinated content (for the nonfluorinated AEBMs, PBIOO was used as matrix polymer and sulfonated PPSU as the ionic macromolecular cross-linker, and for the partially fluorinated AEBMs, F_6_PBI as matrix polymer and SFPE001 as the ionic macromolecular cross-linker (Table 1, Figure 3); (b) the different anion-exchange polymer contents leading to different IECs of the AEBMs; and (c) two different types of PEGs (PEG-diglycidylether with *M*_W_ = 500 Da or 6000 Da) and their amounts in the AEBMs resulting in varying hydrophilicity of the blends. The purpose of these variations was to investigate their impact on important membrane properties such as membrane conductivity, alkaline stability and thermal stability.

In this section, the membrane properties are discussed by first presenting an overview of important membrane properties such as the IEC values, conductivities and water uptake values obtained for the various membranes, followed by a more detailed discussion of the membrane conductivities (Section 2.1.2) and the thermal stabilities using thermogravimetry (TGA) (Section 2.1.3). It was of particular interest during this study to determine how the membrane properties such as conductivity and thermal stability would change during alkaline treatment as both alkaline stability and anion conductivity are the most important prerequisites for the suitability of AEMs for alkaline membrane fuel cells or alkaline membrane electrolysis (see Introduction). TGA was used to determine the alkaline stability of the AEBMs since alkaline degradation would most probably lead to a change in the shape of the TGA traces by anion-exchange group splitting-off and/or backbone degradation due to the attack of the OH^−^ ions.

In Table 1, the most important AEBM data including IEC, conductivity (measured in a 1 N NaCl solution using the in-plane setup (see Section 3.3.2)) and water uptake are listed. As stated previously, the –3C and –4C in the membrane number refer to the three- and four-component AEBMs, respectively, NF refers to AEBMs composed of nonfluorinated blend components, while F stands for AEBMs containing the partially fluorinated F_6_PBI and SFPE001.

The characterization results presented in Table 1 are briefly discussed below.

(a) Influence of quaternized cationic groups:

The TMIm-based AEBMs had significantly higher Cl^−^ conductivities than the NMM- and PMP-based AEBMs. From the low measured IEC of the PMP-based AEBM, it is clear that only a minor portion of the anion-exchange groups was formed under the applied membrane formation conditions which can be traced back to the high bulkiness of PMP.

(b) Calculated vs. measured IECs:

In nearly all cases the measured were higher than the calculated IECs, particularly after the 10 days KOH test (with the exception of the PMP-based 2257 as explained above). In two studies, Aili et al., when investigating the interaction of poly(2,2′-(m-phenylene)-5,5′-bibenzimidazole membranes with KOH [43,44], observed that KOH deprotonates a part of the imidazole-N–H groups, forming imidazole-N^−^–K^+^ ion pairs. Subsequently, the N^−^ anions can form ionic cross-links by electrostatic interaction with the cations of the anion-exchange blend component according to:

PBI-N^−^–K^+^ + Poly-N^+^–Cl^−^ → PBI-N^−^–^+^N-Poly + K^+^–Cl^−^

When determining the IEC, the AEBM that is in the OH^−^ form is immersed in an excess of HCl solution which not only neutralizes the OH^−^ counter ions but also splits the N^−^–^+^N ionic cross-links which consumes additional HCl, leading to the increased measured IEC.

**Figure 5:**
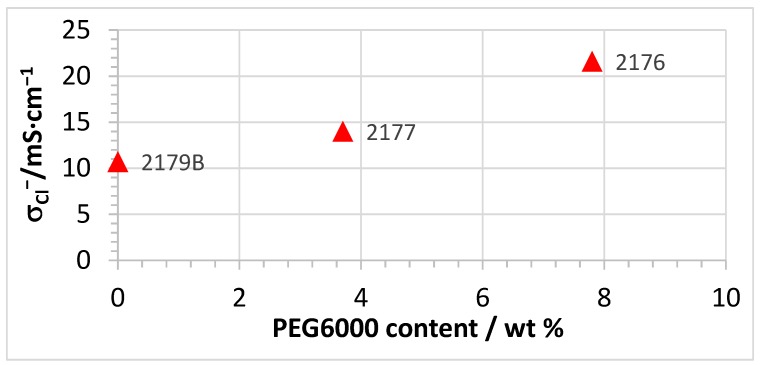
Dependence of Cl^−^ conductivity on the PEG 500 content (measured at room temperature in 1 M NaCl solution) for AEBMs composed of PVBCl, TMIm, PBIOO and sPPSU.

(c) Cl^−^ conductivities of the three- and four-component AEBMs:

The conductivities of the PEG-containing four-component AEBMs are higher than those of the three-component AEBMs, which clearly highlights the positive effect of the hydrophilic PEG blend component on the conductivity. It is particularly noteworthy that already small amounts of PEG (8–10%) lead to a marked conductivity rise. These results confirm literature stating that the introduction of a PEG component into the AEMs increases the anion-conductivity [41,45]. When comparing the conductivities of membranes 2179B, 2177 and 2176, it is clear that the Cl^−^ conductivity increased with increasing PEG content in the AEBM (see also Figure 5).

**Figure 6:**
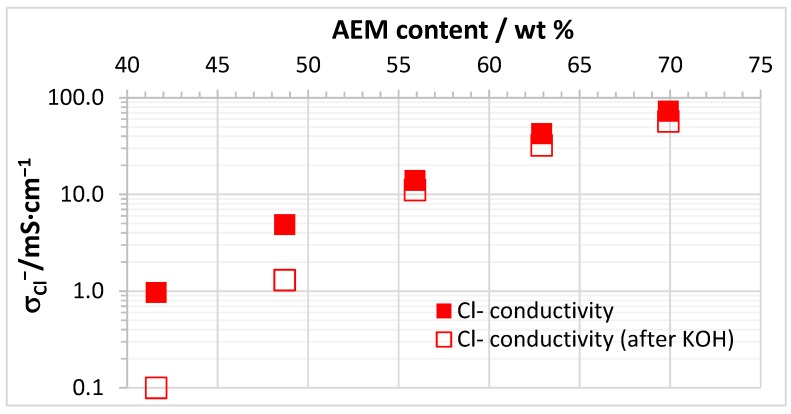
Dependence of Cl^−^ conductivity on the anion-exchange polymer content (measured at room temperature in 1 M NaCl solution) for AEBMs composed of PVBCl, TMIm, PBIOO and sPPSU.

(d) Dependence of Cl^−^ conductivity on the anion-exchange polymer content of the four-component AEBMs:

As expected and reported in the literature (e.g., [46]), the conductivity of the AEBMs increased with increasing anion-exchange polymer content of the blend membranes. The dependence of the Cl^−^ conductivity on the anion-exchange polymer content for AEBMs composed of PVBCl, TMIm, PBIOO and sPPSU is displayed in Figure 6. Interestingly, the decrease of the Cl^−^ conductivity when treated with KOH became less with increasing anion-exchange polymer content, indicating the high OH^−^ stability of the anion-exchange polymers containing PVBCl and TMIm.

(e) Conductivities of partially and non-fluorinated AEBMs with similar calculated IECs:

As can be seen in Table 1, the conductivities of nonfluorinated AEBMs were higher than those of partially fluorinated AEBMs (examples: 2264 (nonfluorinated)/2246 (partially fluorinated; 2261 (nonfluorinated)/2258 (partially fluorinated); 2262 (nonfluorinated)/2259 (partially fluorinated)).

(f)  Water uptake:

In general, the water content of the AEBMs increased with increasing Cl^−^ conductivity.

(g) Membrane weight loss during extraction:

The weight loss remained below 7% for all investigated membranes indicating complete cross-linking within the AEBM blend components. The small weight decrease during extraction is due to the leaching of residual low-molecular tertiary N compounds (they were introduced in 100% excess, as mentioned before) and residual solvents. For two of the membranes (2175 and 2176, see Table 2), the extracted amounts before and after KOH treatment were nearly the same, suggesting alkaline stability of the AEBMs and particularly of the covalent cross-links formed by reaction of the PEGs’ end groups with the PBI-N–H-groups.

(h) Cl^−^ conductivities before and after KOH treatment:

In all cases, the conductivity declined with KOH storage except for the PBIOO-based AEBMs (2175, 2176 and 2190A), where the Cl^−^ conductivity was higher after 10 days of KOH storage. Particularly for 2175 and 2176, the conductivity rise after KOH treatment was significant. The reason for this is unclear, but it can be speculated that these membranes might contain residual KOH physically bound to the PBI blend component by electrostatic interaction (see (b)) which was not completely washed out during the membrane rinsing after the KOH test. Another possible explanation is the possible rearrangement of the microstructure of the membranes during the 10 days KOH treatment. To further investigate this phenomenon, the impedance of some of the membranes were measured using the Scribner Impedance facility which allows the determination of the Cl^−^ conductivity in a vapor state as a function of temperature under constant relative humidity (the amount of relative humidity chosen was 90%). Moreover, the 2176 membrane was subjected to a 30-day exposure to KOH (1 M). The impedance of this membrane was measured after 20 and 30 days of KOH treatment. The results of the detailed impedance investigations of selected AEBMs are presented in Section 2.1.2.

#### 2.1.2. Membrane Conductivities of the AEBMs as a function of Temperature

**Figure 7:**
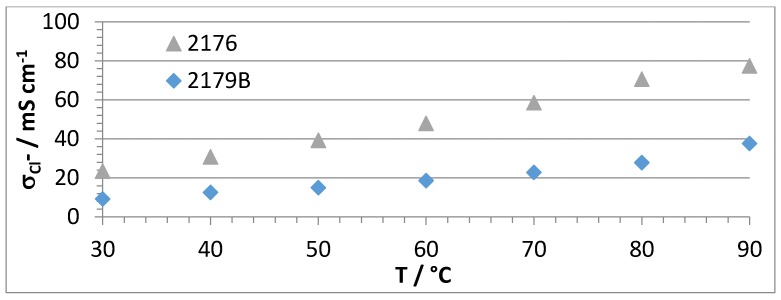
Cl^−^ conductivities of AEBMs with the same calculated IEC, but without PEG (2179B) and with PEG (2176) in dependence of temperature @r.h. of 90% (used PEG: PEG 6000).

**Figure 8:**
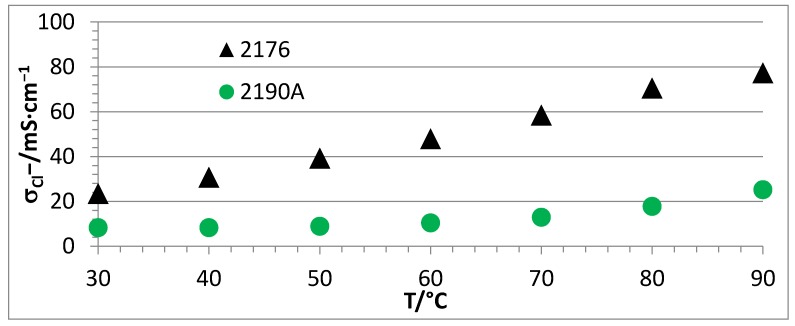
Cl^−^ conductivities of AEBMs with different calculated IEC, but same molar ratio between PBI and PEG component in dependence of temperature @r.h. of 90% (used PEG: PEG 6000; calculated IEC of 2176: 2.3 meq OH^−^/g, and calculated IEC of 2190A: 2.0 meq OH^−^/g).

**Figure 9:**
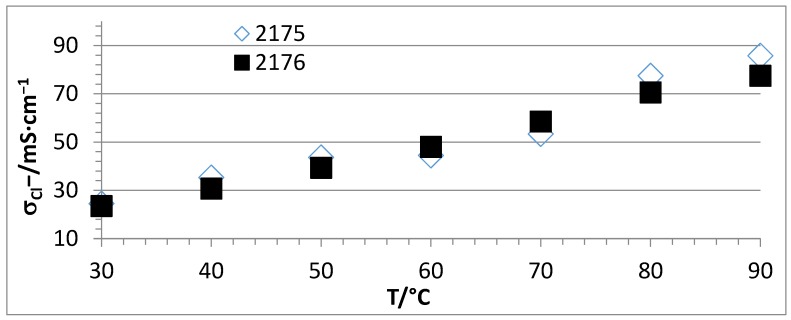
Cl^−^ conductivity vs. T curves of two membranes, 2175 and 2176, with the same composition, but PEGs with different molecular weights (2175: PEG 500, 2176: PEG 6000).

**Figure 10:**
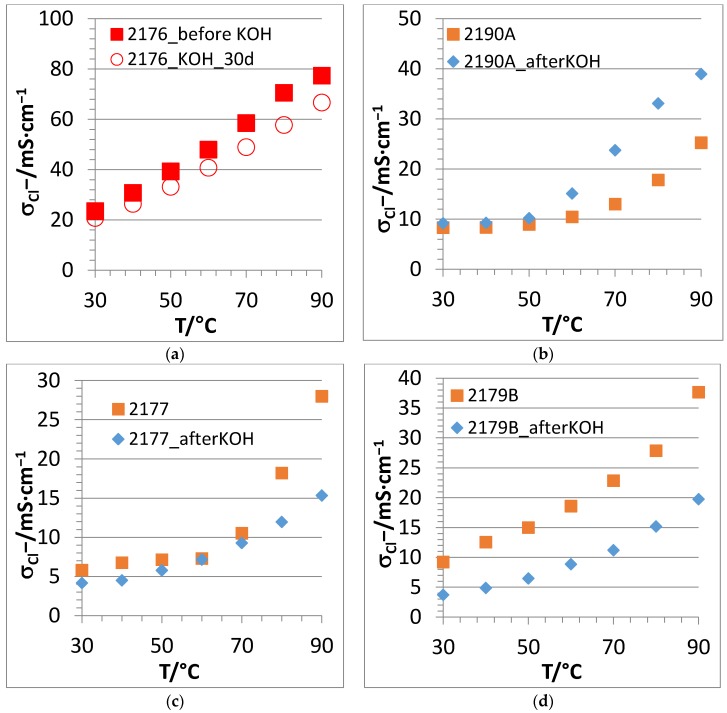

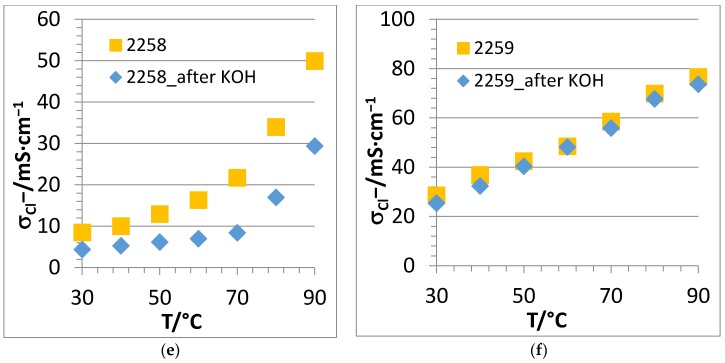
Comparison of Cl^−^ conductivities of several PBIOO-based AEBMs before and after 10 days of KOH treatment (2176: after 30 d KOH treatment) as a function of temperature @90% r.h.: (**a**) membrane 2176; (**b**) membrane 2190A; (**c**) membrane 2177; (**d**) membrane 2179B; (**e**) membrane 2258; and (**f**) membrane 2259.

**Figure 11:**
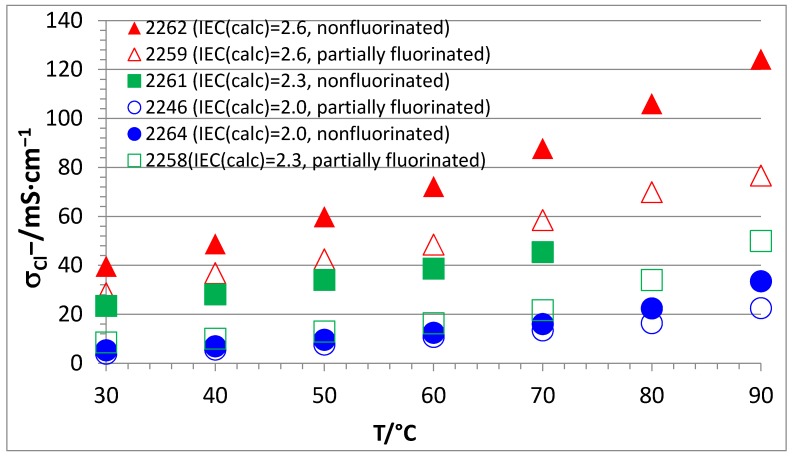
Cl^−^ conductivities of nonfluorinated and partially fluorinated AEBMs.

A selection of membranes were investigated in terms of Cl^−^ conductivity as a function of temperature using the Scribner Membrane Test System (Southern Pines, NC, USA) at a relative humidity of 90% and a temperature range of 30–90 °C. The following comparisons were done:

- Membranes with the same calculated IECs (IEC_calc_ = 2.3 meq/g), but with or without PEGs (NF-AEBM 2179B without PEG, and 2176 with PEG, see Figure 7).
- Membranes with different calculated IECs, but the same PBI:PEG molar ratios (NF-AEBMs 2176 and 2190A, see Figure 8).
- Membranes with the same composition, but different PEG molecular weights (NF-AEBM 2175 with PEG500, and NF-AEBM 2176 with PEG6000, see Figure 9)
- Conductivities of selected NF-AEBMs and two F-AEBMs before and after KOH treatment (2176 (Figure 10a), 2190A (Figure 10b), 2177 (Figure 10c), 2179B (Figure 10d), 2258 (Figure 10e) and 2259 (Figure 10f)).
- Conductivities of NF-AEBMs in comparison with F-AEBMs (membranes 2264/2246, 2261/2258 and 2262/2259 with the calculated IECs_calc_ 2.0, 2.3 and 2.6, respectively, where the IEC variation was obtained by varying the TMIm-quaternized PVBCl content in the blend (Figure 11).

Figure 7 shows that the conductivity was roughly doubled from the membrane without PEG (2179B) to that containing 7.8% PEG (2176) reaching conductivity values of nearly 80 mS·cm^−1^ at 90 °C.

In Figure 8, the strong influence of the IEC on the conductivity is demonstrated. With an IEC_calc_ increase of 0.3 meq/g at 30 °C, the Cl^−^ conductivity was more than doubled. Moreover, due to the higher water uptake of the 2176 membrane (because of its higher IEC), the conductivity increase with temperature was much more pronounced compared to that of the 2190A membrane.

Figure 9 presents the Cl^−^ conductivity vs. T curves of the two membranes 2175 and 2176 with the same composition, but containing PEGs with different molecular weights (2175: PEG 500, 2176: PEG 6000). In Figure 9, it is clear that the molecular weight of the PEG does not have an impact on the conductivity.

In Figure 10, the Cl^−^ conductivity vs. T curves of several of the investigated membranes are compared before and after 10 days of KOH treatment. In the case of the 2176 membrane, the plot was obtained after 30 days of KOH treatment.

The fact that the 2176 membrane lost only 14% of Cl^−^ conductivity after a 30 days KOH treatment (Figure 10a) confirms the excellent alkaline stability of this membrane, which is among the best alkaline stability values reported for anion-exchange membranes to date. When comparing the before/after KOH conductivity results of the 2190A membrane, which comprises a lower calculated (and experimental) IEC than 2176, it is clear (Figure 10b) that this membrane possessed a higher conductivity after 10 days, which was even more pronounced at higher temperatures. This behavior cannot currently be satisfactorily explained, but it could be that low-molecular hydrophilic residues in the membrane, which had not been washed out completely from the membrane during the washing procedure after KOH immersion (particularly KOH which is able to deprotonate the N–H group of the PBI blend compound forming a salt pair Im-N^−^K^+^), might also have contributed to the observed increased conductivity. A similar increase of conductivity with KOH treatment time has been reported for another type of PEG-containing AEM [45].

In contrast, 2177 (with roughly half the PEG content of 2176 (Figure 10c)), and 2179B (without PEG (Figure 10d)) showed lower Cl^−^ conductivities after 10 days of KOH treatment. This behavior is a strong indication that the PEG blend component stabilizes the membrane in an alkaline environment, with this effect increasing with increasing PEG content. However, the origin of this behavior is, as already stated, still unclear and requires a more in-depth investigation in ongoing studies. When comparing the two partially fluorinated AEBMs 2258 (Figure 10e) and 2259 (Figure 10f), it is clear that the membrane possessing the higher anion-exchange polymer content (2259) is more stable in KOH than that having the lower anion-exchange polymer content (2258) as would have been expected.

Figure 11 compares the conductivities of NF- and F-four-component AEBMs for the three calculated IECs of 2.0, 2.3, and 2.6 meq OH^−^/g. It is clear that, for all three different anion-exchange polymer contents, the nonfluorinated AEBMs had higher conductivities than the partially fluorinated AEBMs. This can be ascribed to the higher hydrophobicity of the F-AEBMs leading to lower water uptake values causing lower Cl^−^ conductivities in comparison to the nonfluorinated AEBMs.

#### 2.1.3. Thermal Stability of the AEBMs

A high thermal stability is an important prerequisite for the application of ionomer membranes to those electrochemical applications with working temperatures above room temperature such as fuel cells and electrolysers which should be operated in the T range > 60–80 °C to benefit from improved electrode kinetics at elevated temperatures. The thermal stability of the AEBMs was determined using thermogravimetry (TGA). Of particular interest was whether the shapes of the TGA traces were altered after KOH treatment, indicating membrane degradation.

**Figure 12:**
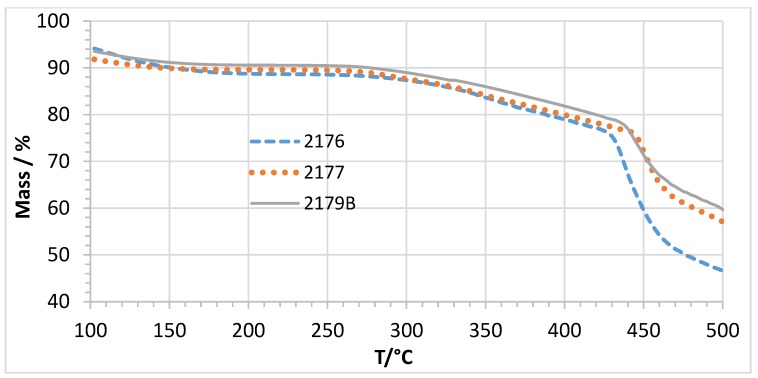
TGA traces of membranes 2176, 2177 and 2179B (different PEG 6000 content).

In Figure 12, the TGA traces of membranes 2176, 2177 and 2179B with similar calculated IEC values, but different PEG contents (see Figure 5), are depicted. It is clear that all displayed membranes possess high and comparable thermal stabilities.

To further elucidate the TGA traces, coupling of the TGA with other spectroscopical techniques can be used. In our group, TGA-FTIR coupling experiments were done to identify the thermal degradation processes during heating of the membranes in the TGA measurement cell [47]. This will be exemplified with the example of membrane 2176 and its blend components.

**Figure 13:**
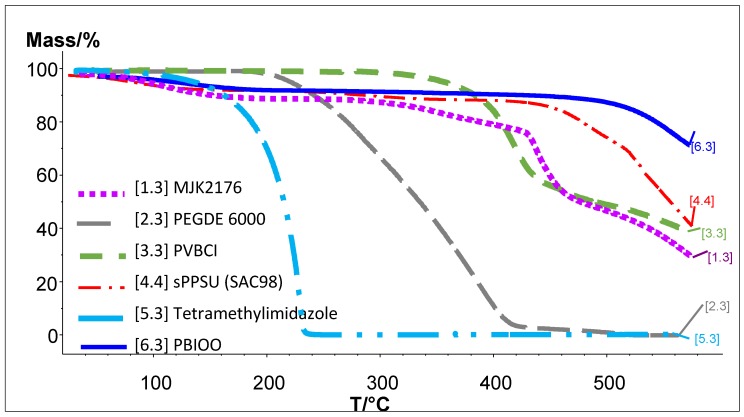
TGA traces of 2176 and its blend components.

In Figure 13, the TGA traces of the 2176 and its blend components are depicted. It can be seen that the blend membrane is more stable than the least stable of the blend components, PEG-diepoxide, which reflects the high chemical reactivity of the epoxide group and the relatively low thermal stability of the poly(ethylene glycol) macromolecular chains. This obviously also indicates the high thermal stability of the blend membrane. The shape of the TMIm TGA trace is similar to a vapor evaporation curve, which means that the TMIm is probably not thermally decomposed during the TGA experiment. This finding was confirmed by the TGA-FTIR coupling experiment where the series spectra showed the signature FTIR spectrum of TMIm.

**Figure 14:**
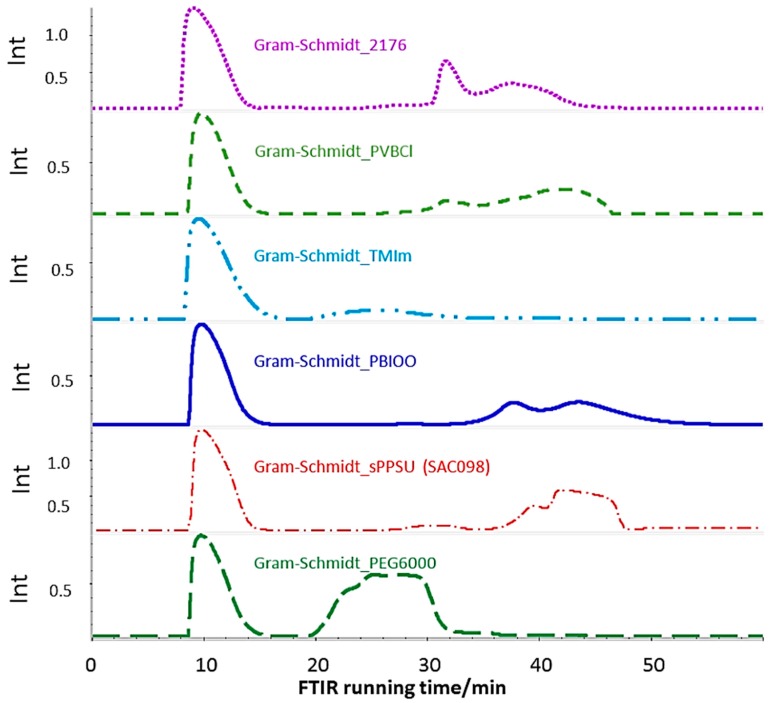
Gram–Schmidt traces of 2176 and its blend components.

**Figure 15:**
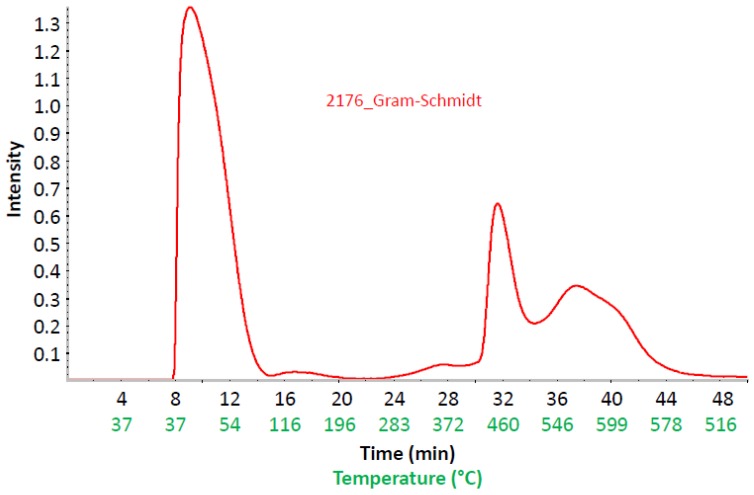
Gram–Schmidt trace of 2176 as intensity vs. FTIR running time and temperature.

Figure 14 shows the Gram–Schmidt traces of the 2176 and its blend components, again confirming that the PEG has the lowest thermal stability among the blend components. In Figure 14, the large peak after roughly 8 min of FTIR running time is the CO_2_ marker signal which allows the assignment of temperatures to the passed time of the TGA-FTIR experiment, or, in other words, the assignment of the temperature to each FTIR spectrum of the gaseous degradation products. The low thermal stability of the PEG-diepoxide blend compound can be confirmed by its Gram–Schmidt trace, i.e., the large peak between 20 and 32 min. This large degradation peak has only a weak echo in the 2176 membrane, showing a slight raise in the 20–32 min. range. It seems that the thermal stability of the PEG blend component was increased by the proposed cross-linking reaction between the epoxide end groups and the imidazole N–H groups of the PBIOO blend component. The high thermal stability of the cross-linked AEBM can be explained not only by the covalent cross-linking reaction between the PEG-epoxide and the PBIOO–N–H, but also by ionic cross-linking between the sPPSU and the basic blend components, the TMIm-quaternized PVBCl and the PBIOO. Figure 15 depicts a more detailed Gram–Schmidt trace of 2176, where the correlation between temperature and FTIR running time is represented. In Figure 15 the large peak after roughly 8 min of FTIR running time is again the CO_2_ marker signal.

**Figure 16:**
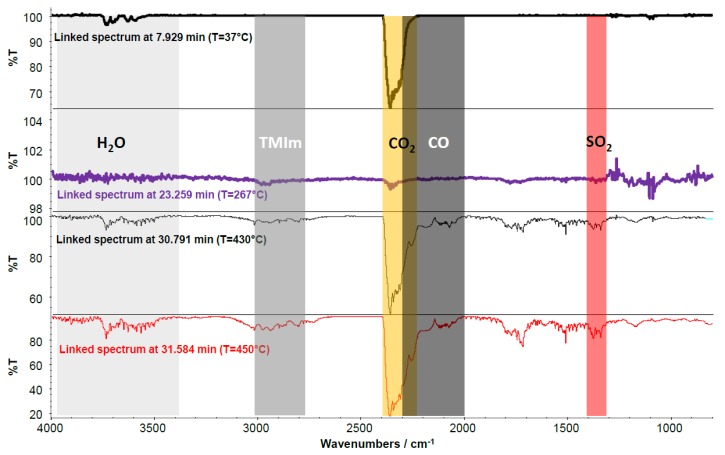
FTIR spectra of the TGA-FTIR experiment of 2176 assigned to *T* = 37, 267, 430, and 450 °C.

The next step of the analysis of the TGA-FTIR coupling results is the assignment of TGA trace steps and Gram–Schmidt trace peaks to the FTIR spectra behind them. In Figure 16, the FTIR spectra of the TGA-FTIR experiments of 2176 assigned to *T* = 37, 267, 430, and 450 °C are presented. The first FTIR spectrum in Figure 16 displays the CO_2_ band (2250–2400 cm^−1^) from the CO_2_ marker signal injected into the TGA cell. At *T* = 267 °C, the following bands were found:

- water evaporating from the membrane(4000 to 3400 cm^−1^)
- onset of CO_2_ development indicating the beginning degradation of the blend membrane, probably originating from the PEG blend component (the TGA-FTIR coupling experiment of pure PEG-diepoxide also showed an onset of CO_2_ formation in the same temperature range).

The FTIR spectrum of the TGA decomposition products at 430 °C indicates an ongoing degradation of the AEBM confirmed by CO_2_ and CO development (CO band from 2000 to 2300 cm^−1^). Moreover, at this temperature, a SO_2_ band (1300–1400 cm^−1^) appeared, indicating the onset of the splitting-off of the sulfonate groups of the sulfonated PPSU blend component [48]. In addition, a broad band appears between 2800 and 3000 cm^−1^, which can be assigned to the TMIm being split off from the quaternized anion-exchange polymer blend component. Water vapor is also observed, probably originating from the thermal decomposition of the AEBM (keeping in mind that the TGA experiments were performed under an oxygen-enriched air which facilitates the thermal oxidation of the organic constituents of the AEBM finally leading to CO_2_/CO and H_2_O formation). The bands between 1700 and 1800 cm^−1^ and between 1100 and 1200 cm^−1^ observed at this temperature probably originated from the decomposition of the PEG blend component as had been observed for the pure PEG-diepoxide.

**Figure 17:**
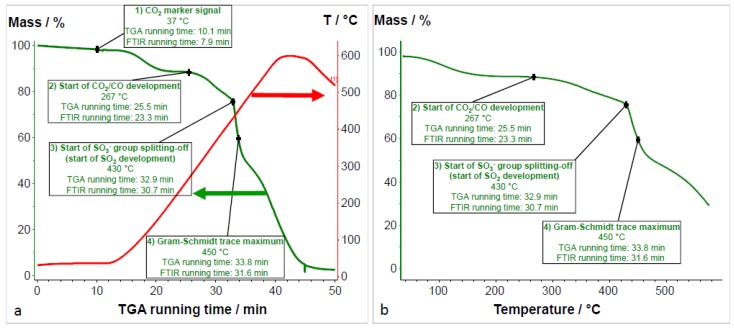
Weight gain vs. TGA running time (**a**) and weight gain vs. temperature (**b**) TGA traces with highlighted points.

The FTIR spectrum of 2176 at 450 °C (where the maximum intensity of the Gram–Schmidt trace was observed) confirms the ongoing degradation of the AEBM. In Figure 17, the weight gain vs. TGA running time (Figure 17a) and weight gain vs. temperature (Figure 17b) TGA traces are presented where the onset of CO_2_ formation, the onset of SO_2_ formation, and the maximum of the Gram–Schmidt trace are highlighted.

The first step of the TGA trace (from room temperature to 200 °C) was due to water vapor evaporation from the water-swollen membrane, as confirmed by the FTIR spectra in this temperature range. Interestingly enough, CO_2_ development was only observed at the beginning of the second stage (from 260 to 430 °C) of the TG vs. T TGA trace. While the second step of the TGA trace was mainly caused by the degradation of the PEG blend component, the weight loss within this step was higher than the PEG content of the membrane (PEG content of 2176 was 7.8%, while the weight loss during the second step was 13.5%). This implies that not only the PEG was degraded during the second step, but also other constituents of the membrane which contributed to the formation of CO_2_ and CO.

**Figure 18:**
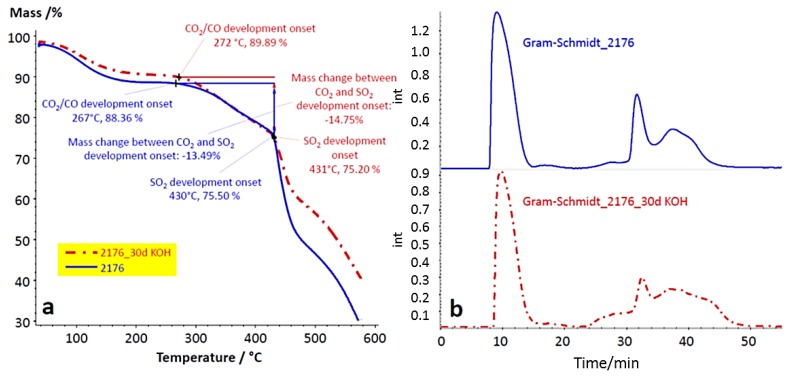
TGA traces (**a**) and Gram–Schmidt traces (**b**) of the 2176 before and after 30 days of KOH treatment.

At the end of the second stage at 430 °C, the onset of both SO_2_ and TMIm was observed, indicating the onset of the splitting-off of ionically-cross-linking anion-exchange and cation-exchange groups which intensified during the third step of the TGA trace. At 450 °C, the maximum intensity of the Gram–Schmidt trace was observed for 2176. The complex FTIR spectrum at this temperature indicates the simultaneous existence of different thermal degradation processes of both the functional groups and polymer backbones of the blend components. Since the alkaline stability of anion-exchange membranes is an important prerequisite for their application in alkaline fuel cells, the comparison of the TGA traces and the TGA-FTIR coupling experiment results is of interest, as the chemical degradation of the AEBMs by the KOH treatment would result in a change of the shape of both the TGA traces and the TGA-FTIR coupling results, where an OH attack might lead to partial splitting-off of anion-exchange groups and possibly also to partial degradation of the polymer backbone of the blend components. In Figure 18a,b, the TGA traces and the Gram–Schmidt traces of 2176 before and after 30 days of KOH treatment are presented.

Figure 18 shows only small differences in both TGA and Gram–Schmidt traces in the *T* range up to 420–430 °C, which implies that the structure of 2176 basically remained intact during the KOH treatment. This is also supported by the conductivity results obtained with 2176, which decreased by only 14%.

**Figure 19:**
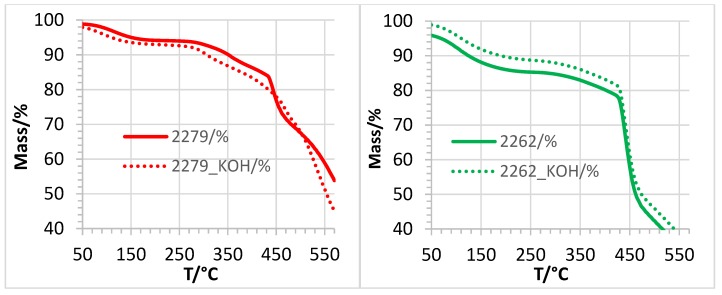
TGA traces of the AEBMs 2262 and 2279 before and after KOH treatment (10 days).

The conductivity results of the AEBMs suggest that the chemical stability of the AEBMs against KOH-induced degradation of the anion-exchange groups increased with increasing anion-exchange polymer content (see Figure 5), which should also reflect in the TGA traces. As an example, the TGA traces of 2262 (calculated IEC = 2.6 meq/g) and 2279 (calculated IEC = 1.4 meq/g) before and after KOH treatment are presented in Figure 19.

From the TGA traces of 2262, it can be seen that the height of the third step of the TGA traces between 420 and 460 °C, where the largest part of the anion-exchange groups was split off (see the analysis of the TGA-FTIR results of 2176), was nearly the same before and after KOH treatment, confirming the significant alkaline stability that had been discussed in Table 1 (conductivity results of 2262). In the case of 2279, the third stage of degradation is much less pronounced after KOH treatment than before, indicating a substantial degradation of anion-exchange groups during the KOH immersion time. This was again confirmed by the 90% conductivity decrease of conductivity by the KOH treatment.

**Figure 20:**
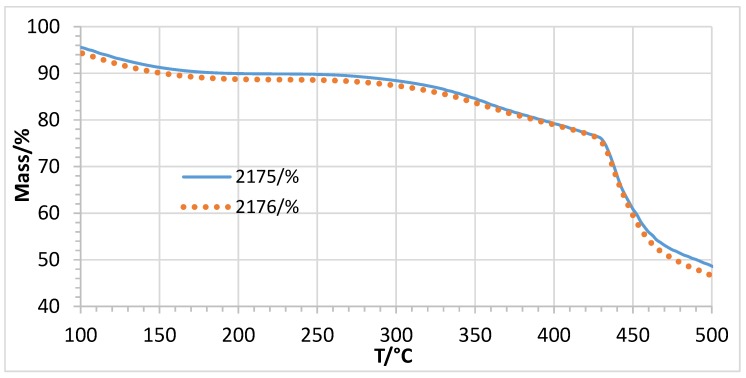
TGA traces of membranes 2175 and 2176 (different PEG molecular weight).

Subsequently, the influence of the molecular weight of the applied PEG-diepoxide on the thermal stability of the AEBMs was determined. In Figure 20, the TGA traces of 2175 (containing PEG500) and 2176 (containing PEG6000) are presented. It is clear that the molecular weight of the applied PEG did not influence the thermal stability.

**Figure 21:**
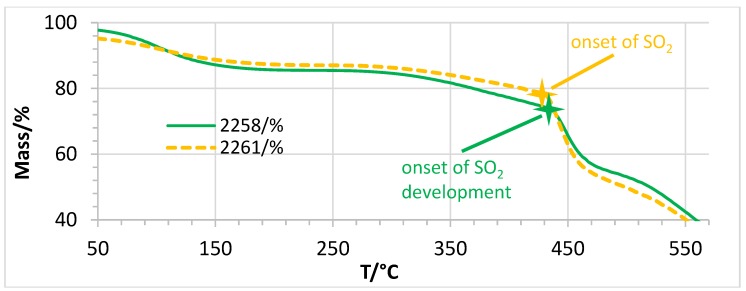
TGA traces of 2258 (partially fluorinated) and 2261 (nonfluorinated).

Finally, the TGAs of nonfluorinated and partially fluorinated AEBMs was compared using the nonfluorinated 2261 and the partially fluorinated 2258 (Figure 21). One can see that the thermal stability of both membranes was comparable. This implies that it is not necessarily advantageous to prepare partially fluorinated anion-exchange membranes, which has the further disadvantage that C–F bonds are not very stable in an alkaline environment as F can be split off by an OH^−^ ion attack in both aliphatic and aromatic C–F bonds [48,49]

## 3. Materials and Methods

### 3.1. Materials

The solvents (DMAc and DMSO) were used as received. The sulfonated polymer (SPPSU098) was synthesized as described earlier [50]. PBIOO was purchased from Fuma-Tech (Bietigheim-Bissingen, Germany). *N*-methylmorpholine (NMM) and 1,2,4,5-tetramethylimidazole (TMIm) were purchased from TCI Chemicals, and 1,2,2,6,6-pentamethylpiperidine (pempidine, PMP) from Manchester Organics. PEG-diglycidyl ether (*M*_n_ = 500 Da, product No. 475696, and *M*_n_ = 6000 Da, product No. 731803, respectively) were obtained from Sigma-Aldrich Germany (Munich, Germany). Poly(vinylbenzyl chloride) (PVBCl), 60/40 mixture of 3- and 4- isomers, was purchased from Sigma-Aldrich, product number 182532. Following the information provided by Sigma-Aldrich, the average molecular weight of PVBCl was: average *M*_n_ ≈ 55,000 Da, average *M*_w_ ≈ 100,000 Da (determined by GPC/MALLS according to the Sigma-Aldrich website).

### 3.2. Membrane Preparation and Posttreatment

All blend components (except for the PEGs in the four-component AEBMs) were separately dissolved in DMAc or DMSO as 5%, 10%, 15% or 20% solutions, before being added in specific ratios to prepare the AEBMs (see Table 1 for composition of blends). For the four-component AEBMs, the different PEG-diglycidyl ethers (mol. wt.: 500 and 6000 Da) were added in pure form. To accelerate the dissolution rate of the 6000 Da PEG, the temperature was increased to 60 °C. After dissolution of the PEGs, the tertiary amines were added as 33.3% solutions in DMAc (TMIm) or in pure form (NMM, PMP) in 100% excess in terms of the amount of chloromethyl groups in the blend solution. After mixing, the blend solutions were cast onto glass plates and the solvent was evaporated in a convection oven at *T* = 140 °C for 2 h. After immersion in deionized water, the membranes came off the glass plates. The membranes were posttreated as follows:

1. The membranes were immersed in 10% ethanolic solutions of different tertiary amines. Most of the membranes were posttreated with 1,2,4,5-tetramethylimidazole (TMIm) to complete the quaternization reaction.
2. The membranes were immersed in 10% NaCl solution for 48 h at 90 °C.
3. The membranes were washed thoroughly with DI water and stored in DI water at 60 °C for 48 h.
4. The membranes were soaked in an aqueous 1 M KOH solution at 90 °C for 10 days. Some of the membranes were stored in a 1 M KOH solution at 90 °C for 20 and 30 days, respectively.
5. To determine the effectiveness of the reaction of the PEG’s epoxy end groups with the N–H groups of the PBI, a 10 wt % DMSO solution of pure PBI (80 wt %) was mixed with the PEG (PEG500, 20 wt %), before casting a membrane from this mixture. After membrane formation, the blend membrane was extracted with DMAc to determine the extent of cross-linking within the blend. According to the results the DMAc residual after extraction was nearly 100%, proving the completeness of cross-linking of the PBI with the epoxide end groups which confirms the covalent cross-linking of PBI with bisphenol A-bisepoxide and other bisepoxide compounds [51].

The compositions of the AEBMs are listed in Table 2.

### 3.3. Membrane Characterization

#### 3.3.1. Ion-Exchange Capacity (IEC)

The IEC was determined as described in a recent paper of our group [38].

#### 3.3.2. Ionic Conductivity

The ionic Cl^−^ conductivity of the membranes was determined via impedance spectroscopy (EIS). For the measurement, two different setups were used: for series impedance measurements an in-plane self-constructed impedance cell was used which allowed EIS measurements in a liquid state (1 N NaCl) at room temperature (25 °C). The cell was connected to a Zahner elektrik IM6 impedance spectrometer (Kronach, Germany). The impedances of the membranes were investigated in a frequency range of 200 Hz–8 MHz (amplitude: 5 mV). Details of the measurement are described in [38]. The ohmic resistance obtained from EIS measurement was converted into conductivity using the following equation:

(1) $$\sigma =\frac{1}{R_{sp}}=\frac{d}{R\times A}$$

where, σ: Conductivity, mS·cm^−1^; *R_sp_*: resistivity, Ω·cm; *d*: thickness of the membrane, cm; *R*: ohmic resistance, Ω; *A*: electrode area, cm^2^.

The second impedance setup used for selected membranes was a Membrane Test System (MTS 740, Scribner Associates Inc., Southern Pines, NC, USA), which allows through-plane impedance measurements in a vapour phase as a function of temperature and relative humidity independently from each other, connected to a Solartron 1260 impedance analyser. Details of the measurements are described in a previous paper [40].

#### 3.3.3. Gel Content

The gel content of the membranes, which is a measure for the extent of cross-linking within the AEBMs, was determined by extraction with 90 °C hot DMAc, as described in [40].

#### 3.3.4. Water Uptake

The water uptake of the membranes was determined by immersion in water at the respective temperature until equilibration had been reached, as described in [40].

#### 3.3.5. Thermal Stability

Characterization of the thermal stability of the membranes in the chloride form was performed by thermogravimetry (TGA, Netzsch, model STA 499C, Netzsch, Selb, Germany) at a heating rate of 20 °C·min^−1^ under O_2_-enriched O_2_/N_2_ atmosphere (65%–70% O_2_) which was coupled with a FTIR for FTIR analysis of the gaseous thermal degradation products.

#### 3.3.6. Alkaline Stability

Due to the application of AEMs in electrochemical systems such as alkaline membrane fuel cells or alkaline membrane electrolysis where the AEMs are in the hydroxide form, the stability in strongly alkaline environments is the most important prerequisite for determining their suitability for these processes. Consequently, the stability of the AEBMs was investigated under harsh conditions, i.e., in 1 M KOH at a temperature of 90 °C for 10 days, or even in one case (membrane 2176) for up to 30 days. Membranes were characterized before and after the stability tests using Cl^−^ conductivity, IEC and thermal stability.

## 4. Conclusions

In this study, novel AEBMs based on poly(vinylbenzyl chloride) quaternized with different tertiary basic N compounds, PBIOO or F_6_PBI as the stabilizing matrix polymer and a sulfonated poly(ethersulfone) or a partially fluorinated sulfonated aromatic polyether as ionic macromolecular cross-linker were synthesized and characterized. Epoxide-end-functionalized poly(ethylene glycol)s were introduced into the AEBMs as an additional hydrophilic microphase, leading to a significant increase in the chloride conductivity of the AEBMs. In addition, the introduction of the PEG also led to a marked improvement of the stability of the AEBMs in an alkaline environment. It seems that the presence of the PEG led to a microphase-separation of the AEBMs, which, with high probability, facilitated the clustering of the anion-exchange groups within the AEBM microstructure thereby contributing to the observed conductivity increase of the PEG-containing AEBMs. The thermal stability of the novel AEBMs was excellent. The favorable properties determined for the novel AEBMs make them promising candidates for several electrochemical applications such as redox-flow batteries, alkaline membrane fuel cells and alkaline membrane electrolysers.

## Figures and Tables

**Table 1:** Physico-chemical properties of three- and four-component AEBMs.

Membrane (No.)	IEC_calc_ (meq OH^−^/g)	IEC_exp_ (meq OH^−^/g)	IEC_exp_ * (meq OH^−^/g)	Weight Loss ** (%)	σ_Cl_^−^ (S/cm)	σ_Cl_^−^* (S/cm)	WU (%, RT)	WU (%, 90 °C)
2256_3C_NF	2.3	2.9	3.17	2.4	8.2	1.1	49	51
2257_3C_NF	2.3	0.39	0.45	7.3	0.17	0.12	7	8
2179B_3C_NF	2.3	2.5	2.64	5.8	10.7	15.9	63	100
2223_3C_NF	2.0	2.97	3.15	2.5	10.8	5.4	56	79
2177_4C_NF	2.3	2.2	2.44	3.4	14.0	10.6	36	42
2175_4C_NF	2.3	2.92	2.96	2.4/0 ^§^	29.3	72.7	367	n.m.
2176_4C_NF	2.3	2.79	2.84	2.6/3 ^§^	21.6	69.9	370	436
2190A_4C_NF	2.0	2.1	2.73	4.1	14.3	16.3	67	120
2261_4C_NF	2.3	2.2	n.a.	0.6	42.8	32.2	132	212
2262_4C_NF	2.6	n.a.	3.53	2.1	72.9	56.9	242	377
2264_4C_NF	2.0	3.07	2.93	0	14.0	11.1	71	90
2265_4C_NF	1.7	3.07	3.04	0	4.9	1.3	41	47
2279_4C_NF	1.4	2.89	3.04	0	0.97	0.1	28	28
2267_4C_NF	2.3	2.92	2.84	0	51.7	60.5	290	371
2246_4C_F	2.0	2.23	2.38 ***	2.1	3.9	4.4 ***	29	34
2258_4C_F	2.3	2.42	2.53	1.9	26.4	16.1	77	91
2259_4C_F	2.6	2.41	2.74	0.9	45.4	46.8	157	258

* After 10 days in 10% KOH at 90 °C; ** after extraction with DMAc at 90 °C; *** after 30 days in 10% KOH at 90 °C; ^§^ weight loss after 10 days of KOH storage.

**Table 2:** Composition of blend components for AEBM preparation.

Membrane Type * (No.)	IEC_calc_ (meq OH^−^/g)	Type amine	PVBCl-AEM (%)	PBI (%)	S-Polymer (%)	Type PEG (Da)	PEG (%)
2256_3C_NF	2.3	NMM	58.3	PBIOO/32.8	SPPSU **/8.9	-	-
2257_3C_NF	2.3	PMP	70.0	PBIOO/23.6	SPPSU/6.4	-	-
2179B_3C_NF	2.3	TMIm	63.5	PBIOO/28.7	SPPSU/7.8	-	-
2223_3C_NF	2.0	TMIm	54.9	PBIOO/38.4	SPPSU/6.8	-	-
2177_4C_NF	2.3	TMIm	62.9	PBIOO/26.0	SPPSU/7.4	6000	3.7
2175_4C_NF	2.3	TMIm	63.5	PBIOO/20.9	SPPSU/7.8	500	7.8
2176_4C_NF	2.3	TMIm	63.5	PBIOO/20.9	SPPSU/7.8	6000	7.8
2190A_4C_NF	2.0	TMIm	54.9	PBIOO/27.9	SPPSU/6.8	6000	10.4
2261_4C_NF	2.3	TMIm	62.9	PBIOO/24.1	SPPSU/7.4	500	5.6
2262_4C_NF	2.6	TMIm	69.9	PBIOO/19.5	SPPSU/6.0	500	4.5
2264_4C_NF	2.0	TMIm	55.9	PBIOO/28.6	SPPSU/8.8	500	6.6
2265_4C_NF	1.7	TMIm	48.7	PBIOO/33.4	SPPSU/10.3	500	7.7
2279_4C_NF	1.4	TMIm	41.6	PBIOO/41.6	SPPSU/11.7	500	8.8
2267_4C_NF	2.3	TMIm	65.2	PBIOO/18.2	SPPSU/12.3	500	4.2
2246_4C_F	2.0	TMIm	56.6	F_6_PBI/28.98	SFPE ***/7.8	500	6.7
2258_4C_F	2.3	TMIm	63.5	F_6_PBI/24.4	SFPE/6.5	500	5.6
2259_4C_F	2.6	TMIm	70.5	F_6_PBI/19.7	SFPE/5.3	500	4.5

* Abbreviations: 3C_NF: three-component AEBMs, containing the nonfluorinated blend polymers PBIOO and sPPSU; 4C_NF: four-component AEBMs, containing the nonfluorinated blend polymers PBIOO and sPPSU; 4C_F: four-component AEBMs, containing the partially fluorinated blend polymers F_6_PBI and SFPE001; ** SPPSU: sulfonated poly(phenylene sulfone); *** SFPE: sulfonated partially fluorinated aromatic polyether; for structures of the sulfonated polymers, see Figure 3.
